# Metabolomic profiling to detect different forms of beef fraud using rapid evaporative ionisation mass spectrometry (REIMS)

**DOI:** 10.1038/s41538-022-00125-7

**Published:** 2022-01-27

**Authors:** Kelsey Robson, Nicholas Birse, Olivier Chevallier, Christopher Elliott

**Affiliations:** 1 ASSET Technology Centre, Institute for Global Food Security, School of Biological Sciences, Queen’s University Belfast, 19 Chlorine Gardens, Belfast, BT9 5DL Northern Ireland UK; 2 ABP Food Group, Bishops Court, Solihull Parkway, Birmingham, B37 7YB UK; 3 Avignon Universite, Maison de la Recherchem, Pole Structure et Infrastructure de Recherche Partagée, Campus Jean-Henri Fabre, Bâtiment A - Bureau A104, 301 rue Baruch de Spinoza BP 21239, 84911 Avignon cedex 9 Avignon, France

**Keywords:** Mass spectrometry, Lipids

## Abstract

Organic food fraud is a significant challenge in the food testing sector—high price premiums, ease of access to produce to be relabelled and difficulties in developing testing strategies that can detect such frauds make organic foods particularly attractive and thus highly vulnerable to fraud. Samples of conventional and organic cattle taken across meat plants in Ireland and the United Kingdom, consisting of the neck (supraspinatus), rump (gluteus), and shin (flexor carpi radialis) regions of the carcass were analysed using a high resolution time-of-flight based rapid evaporative ionisation mass spectrometry (REIMS) system. The resulting untargeted lipidomic data (m/z 600–1000) was used to generate PCA-LDA models for production system and for muscle type, for these models, it was found that the production system model could differentiate organic from conventional beef with an accuracy of 84%, whilst the muscle type model could identify the cut of meat with a 98% accuracy; additionally, samples can be assessed against multiple models simultaneously, reducing analysis time and sample numbers. The use of REIMS showed considerable promise in its ability to detect different forms of meat fraud; its accuracy in differentiating organic from conventional beef is superior to stable isotope ratio mass spectrometry, with the added advantages of substantially shorter analysis times and lower sample analysis costs. The ability to rapidly confirm the cut of meat also demonstrates the potential of REIMS to concurrently determine multiple aspects of beef authenticity in a close to real time analysis.

## Introduction

Between 2010 and 2017, thousands of consumers in the United States of America who were paying a premium for organic food were deceived^1,2^. Four Midwestern farmers had falsely labelled conventionally grown grains as organic and sold them to organic meat and meat product procedures, resulting in farmers feeding conventional feed to their organic cattle and selling meat and animal products to producers and consumers as organic when they were not fed organic feed and therefore not reared by organic standards. This is one example of organic meat fraud. Organic fraud in its totality occurs when a product is labelled organic yet has not been produced by organic standards^3^. Organic foods are among the most vulnerable to fraud, mainly due to how easily this type of fraud can be committed and how difficult it is to detect^4–6^.

Despite regulations and certifications for organic labelling and livestock production, it is difficult to ensure that products on the market are truly organic^7^. Therefore, robust analytical methods are required to support the verification processes. Various techniques have been investigated; however, a conclusive method has not yet been found. Regulations concerning different cuts are even more opaque, with considerable leeway being given on what names different cuts can be given, with further variations between different markets increasing confusion further.

Technologies such as stable isotope ratio analysis (SIRA) have been able to indicate if meat may be organic by detecting isotope ratios that are more consistent with artificial fertilisers and crop protection products; however, it cannot conclusively distinguish between organic and conventionally produced meat^8–10^. SIRA testing is expensive and requires substantial time-consuming sample preparation, therefore, new technologies must be explored to provide testing which is rapid and affordable.

This study involved the collection of samples of meat from conventional and organically reared cattle carcasses, using different muscle types (meat cuts) from those carcasses. The lipid composition of the muscle samples was analysed using a bipolar meat probe connected to a rapid evaporative ionisation mass spectrometry (REIMS) ion source mounted to a high-resolution quadrupole time-of-flight mass spectrometer (HR-QToF-MS).

The resulting spectra were used to generate PCA and PCA-LDA chemometric models, making use of the glycerophospholipids and sphingolipids found in the mass range m/z 600–1000, against which unknown samples could be analysed to determine the overall accuracy of the REIMS based analysis approach.

Organic beef production in the European Union (EU) must follow Council Regulation (EC) No 834/2007. This regulation sets out standards for producing and labelling organic products throughout the EU. It specifies that to produce organic cattle, a farm must be registered with an organic control body (such as Soil Association Certification Ltd, Organic Farmers & Growers CIC, or the Irish Organic Association), and the production system adopted must meet the organic standards specified by that body^11^.

Each control body has its own set of standards that differ slightly. Key aspects which are constant around the EU include:

- Feedstuffs must be produced and certified to organic standards
- At least sixty percent of the diet must come from organic forage
- Genetically modified (GM) animal feed is banned under organic standards
- Veterinary medicines and antibiotics cannot be used as a preventative measure but should treat illness or injury.
- Withdrawal period of any veterinary medicines and antibiotics must be twice the stated withdrawal period for conventionally produced cattle.

Currently, organic beef production primarily relies on certification to prove compliance; however, SIRA has shown promise to distinguish organic from conventional beef^12,13^. Schmidt et al. found significate differences between conventional and organic Irish beef combining carbon, nitrogen, and sulphur isotopic composition (MANOVA, *F*3,28 = 10.3, *P* < 0.001)^12^. Conventional Irish beef had a less negative and more variable ^13^C value (−24.5‰ ± 0.7‰) than organic beef (−26.0‰± 0.2‰). However, it was later found that ^13^C increases in conventional beef between December and June, which dilutes the results of Schmidt et al.^12–14^. SIRA’s success using carbon composition is mainly reliant on a higher proportion of fresh or preserved grass in organic cattle compared with maize and concentrate in conventional cattle^14^.

This approach presents several risks of false results; the use of organic maize or concentrate feeds would give a carbon composition more in keeping with conventional production, whilst conventional production making use of more grass feeds would give a carbon composition more in keeping with organic production.

Levels of ^15^N/^14^N also indicated organic status as ^15^N pathways in conventional beef had been found higher than in organic due to the mineral content in fertilisers^12,13,15^. However, ^15^N pathways can also be found in organic fertilizers, although in lower amounts, whilst dietary differences such as higher legume content in an animal’s diet can also affect ^15^N pathways^12^. Organic beef had also been found to have slightly more ^34^S (7.9‰ ± 0.6‰) compared to conventional (7.2‰ ± 0.4‰) beef. This may be due to organic or mineral fertiliser or feed supplements, but it is not known with any degree of certainty^12,16^.

Other than SIRA, fatty acid profiles might prove useful for the authentication of organic meat as feeding regime has an effect on the lipid profile of the meat. Multiple studies on fatty acids in relation to organic fraud have been reviewed by Capuano et al. (2012). However, no method has yet to be proven as a robust detection method for organic fraud related to beef, as with stable isotope analysis, heavy grass feed in conventional production or heavy maize feed in organic production can cause confusion in assessing the production systems involved. The difference in lipid profile is thought to be a useful method of differentiating different cuts and identifying frauds where a costly premium cut is replaced by a lower quality, lower cost cut, in a mislabelling or substitution type fraud.

The presence of veterinary medicines and antibiotics in organic cattle can assist in determining whether a product is likely organic or not, with the use of treatments only being permitted in the event of illness. Residue tests may not be fully able to prove the organic status of animals but can still be a useful tool in proving that organic standards were not adhered to when rearing the animal in question^17^.

REIMS is a type of ambient mass spectrometry (AMS) that has shown promise in food fraud detection, particularly in the area of meat and fish products^18–21^. It operates using an electrosurgical knife, probe, or laser that produces an aerosol when cutting or burning into a tissue sample. The aerosol is taken from the sample through a transfer line, combined with a solvent containing the reference mass solution, and introduced into the ionisation source which is mounted on the front of the mass spectrometer. The ionisation source consists of a heated Kanthal coil collision impactor assembly in-line immediately adjacent to the instrument orifice which causes rapid ionisation of the incoming smoke and solvent mixture^22^. REIMS has been employed in several food fraud studies, initially being demonstrated for speciation and breed of meat, subsequently, this capability in detecting fraud was extended to fish, the detection of additives in minced meat, the detection of adulterants in minced, and most recently, the differentiation between organic and conventional poultry^19–21,23,24^. REIMS analysis is well suited to the meat, poultry, and fish sectors as it requires no sample preparation, generally takes only a few seconds, and can be used by non-specialised personal. REIMS was more recently demonstrated in a commercial slaughterhouse environment for the detection of boar-taint^18^.

## Results

The results show that REIMS is capable of identifying the individual cuts of meat with a very high degree of accuracy, approaching 100% when potentially mislabelled samples were removed from the model. The performance in correctly identifying conventional and organic production systems was initially less impressive, with cross-validation percentages below 60%. However, with increasing numbers of samples, the cross-validation percentage reached 84%.

The modelling performance for the simultaneous determination of cut and production system was less successful and cross-validation performance was also varied between 70 and 75%; this approach was not pursued further, the alternative would be to use a hierarchical approach with the meat cut being determined first, and then the production system being assessed.

### Distinguishing a cut of beef

**Fig. 1:**
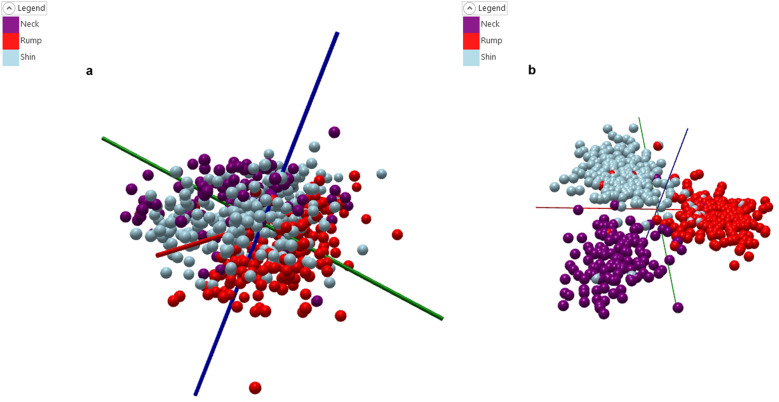
Meat cut models. **a** PCA plot for the meat cut model, with clear groupings shown for neck, rump, and shin cuts. **b** PCA-LDA plot for the meat cut model, with clear separation shown between the three classes, but also showing some mislabelling present within the dataset.

Figure 1a shows the PCA plot for the samples taken from the different areas of the carcass (shin, rump, and neck). The PCA plot clearly shows the separation of the cuts into three distinct groups, following which Fig. 1b, a PCA-LDA model was generated.

The cross-validation for this model (Table 1) was 88.5%, and there was one outlier. It was observed during this initial modelling that several samples appeared to be mislabelled, upon further investigation it became clear that the three cuts from each carcass had, on a number of occasions, become incorrectly labelled, and accordingly these incorrectly labelled samples were removed from the model.

**Table 1:** Leave-20%-Out cross-validation results for the uncorrected meat cut model.

Group	Number of spectra	Number of passes	Number of failures	Number of outliers	Correct classification rate (excluding outliers)	Correct classification rate (including outliers)
Total	561	496	65	1	88.5%	88.5%

**Fig. 2:**
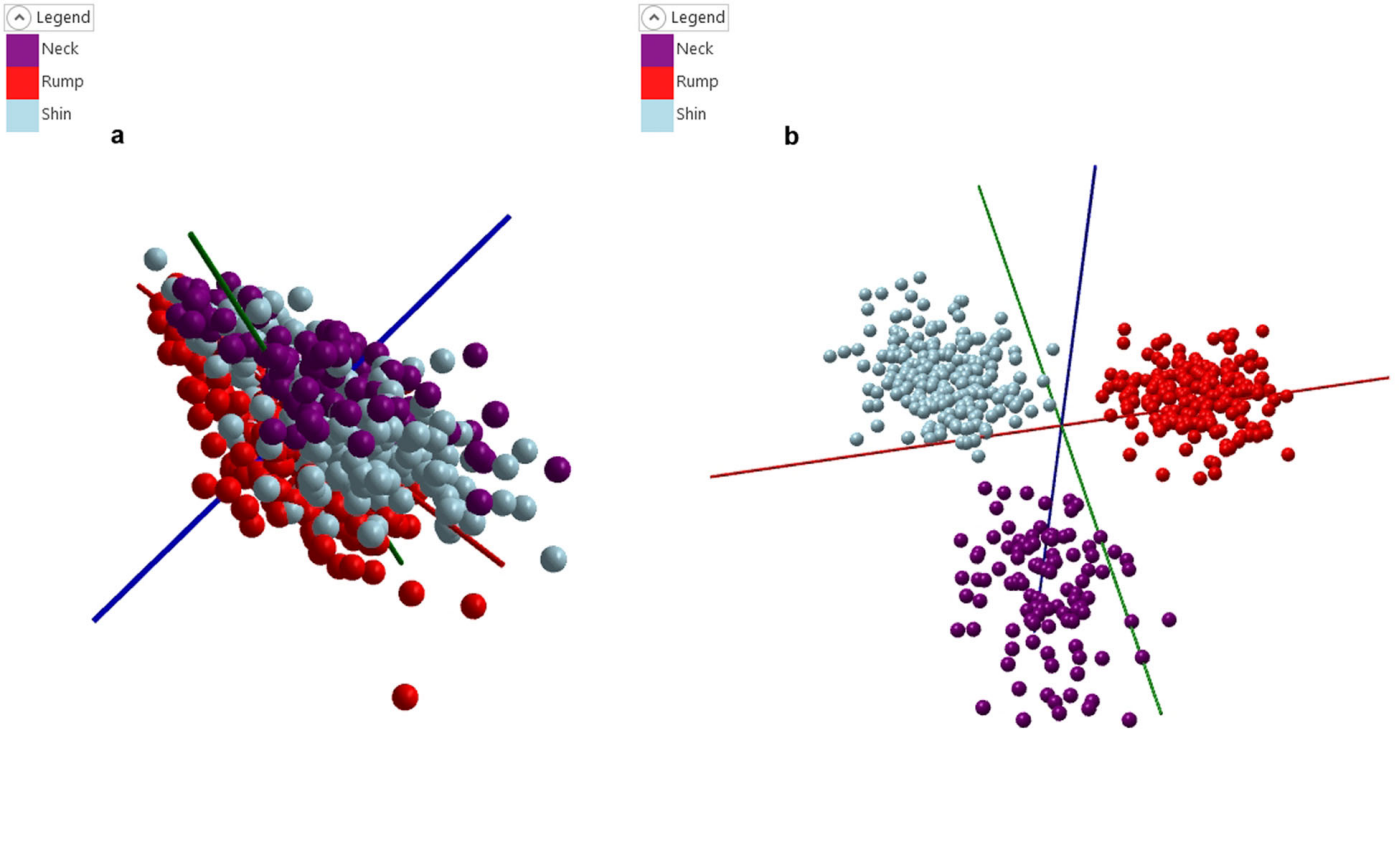
Corrected meat cut models. **a** PCA plot for the corrected meat cut model, with clear groupings shown for neck, rump, and shin cuts. **b** PCA-LDA plot for the corrected meat cut model, greater separation between the three groups can be seen in comparison to Fig. 1b.

The corrected model Fig. 2a shows the PCA plot, again even more clearly it can be observed that there is a clear separation between each of the three cuts. The corrected PCA-LDA model (Fig. 2b) also shows a clear separation of the three groups. The cross-validation for this model (Table 2) is 98.2% with no outliers and only nine failures.

**Table 2:** Leave-20%-Out cross-validation results for the corrected cut model.

Group	Number of spectra	Number of passes	Number of failures	Number of outliers	Correct classification rate (excluding outliers)	Correct classification rate (including outliers)
Total	498	489	9	0	98.2%	98.2%

The strong performance of this model can be attributed to the significant differences in both lipid profile and relative intensities when reviewing the fingerprints for the three different cuts of meat samples. Figure 4a, b shows the two linear discriminatory components of the three cut model with clear variations in both lipid profile and relative intensity.

### Production system

**Fig. 3:**
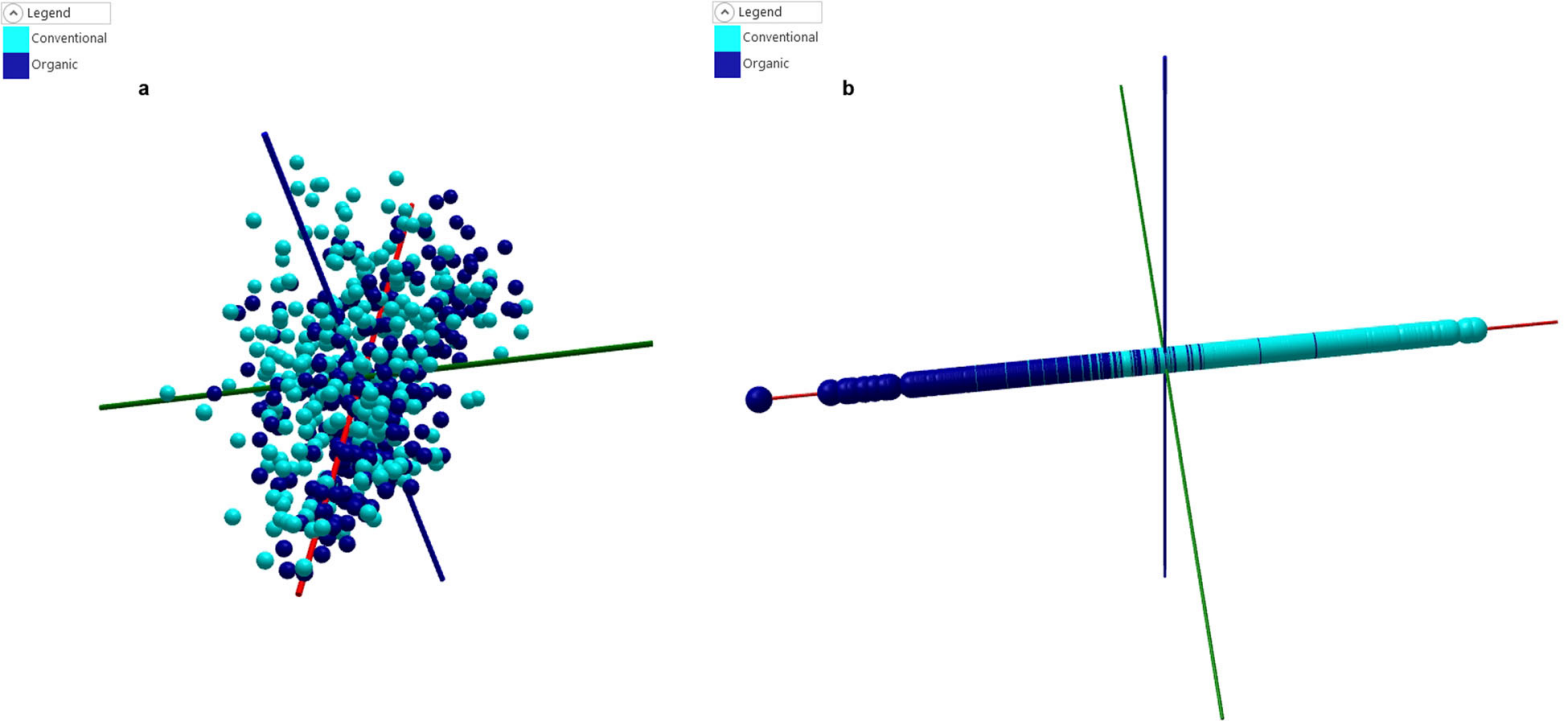
Production system models. **a** PCA plot for the production system model, with weak evidence of grouping by the organic and conventional groups. **b** PCA-LDA plot for the production system model, again with weak evidence of grouping by the organic and conventional groups and no clear separation between the two groups.

Figure 3a shows the PCA plot for the different production systems being examined within the model, in comparison with the meat cut modelling, there is only very weak grouping by production system to be observed within the model. This is further evidenced in the discriminatory PCA-LDA model (Fig. 3b), which shows a relatively low degree of separation, and no clear separation between the two groups.

This PCA-LDA model demonstrated a cross-validation performance of 83.9%, with 90 samples out of 561 being incorrectly identified. These were 53 conventional samples being misclassified as organic and 37 organic samples being misclassified as conventional (Table 3). There was one outlier that could not be identified as either organic or conventional.

Cross-validation performance of the model improved as successive rounds of sampling were undertaken, and decreased as samples with incorrect cut data were removed from the model. This strongly suggests the removed samples whilst the cut was incorrectly labelled, the production system was correctly labelled as organic or conventional. The correct classification rate for organic or conventional production systems in the corrected cut model reduced to 75.4%.

**Fig. 4:**
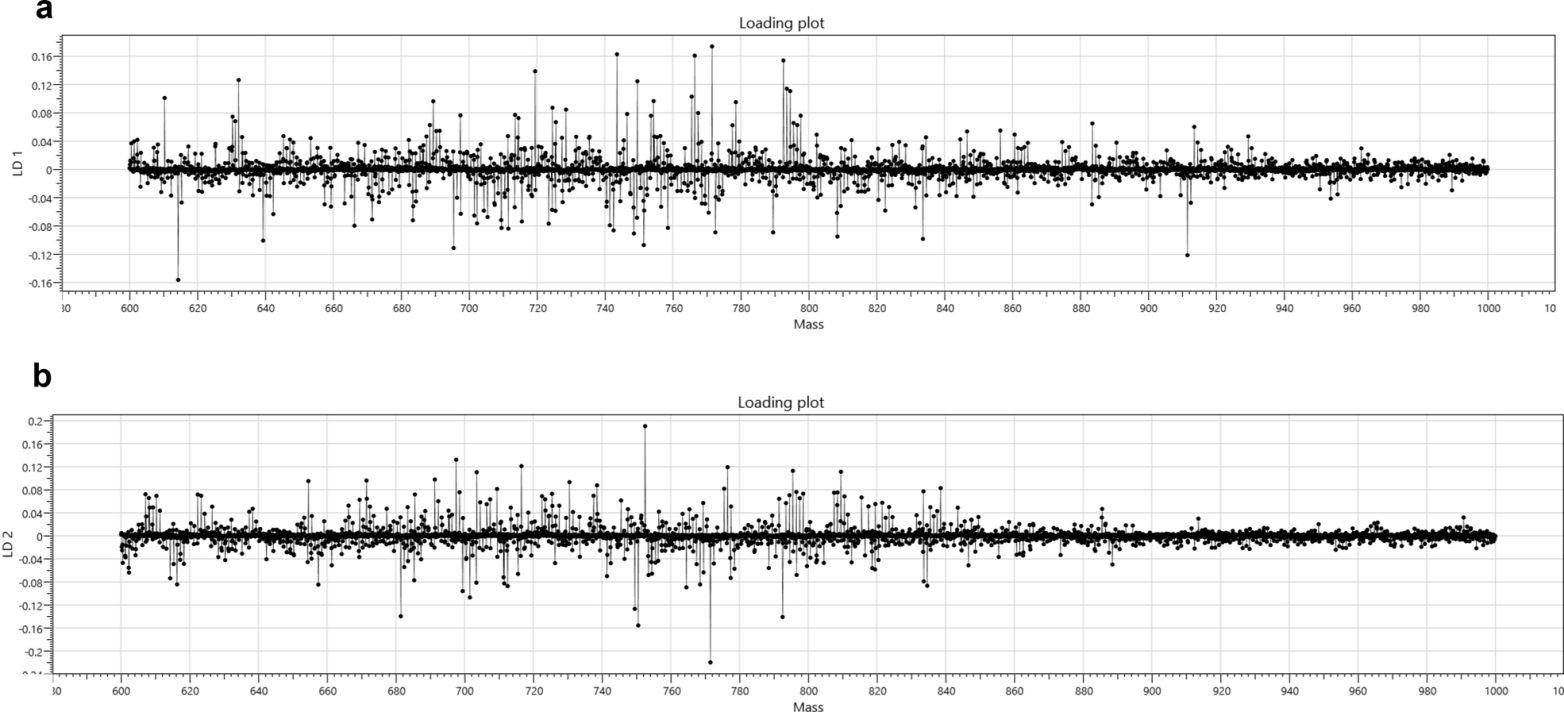
Meat cut loading plots. **a** Loading plot showing discreet differences in lipids for Linear Discriminant 1 in the meat cut model. **b** Loading plot showing discreet differences in lipids for Linear Discriminant 2 in the meat cut model.

**Fig. 5:**
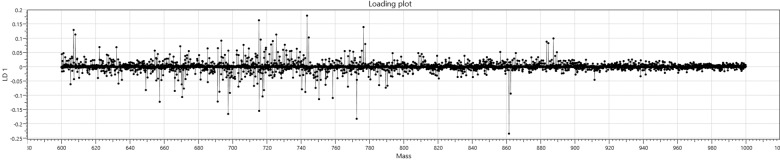
Production system loading plot. Loading plot showing discreet differences in lipids for Linear Discriminant 1 in the production system model.

The limited performance of this model can be attributed to fewer and less intense differences in the lipid profiles of the organic and conventionally produced beef (Fig. 5) which stands in contrast to the more significant differences in lipid profile and intensity differences between cuts of beef (Fig. 4a, b).

### Distinguishing production system and cut of beef

It was considered possible that the lipid differences which resulted in successful cut identification were also responsible for the less successful production system identification, accordingly, three individual models based on the individual cuts were generated to determine whether the production system could be identified from each specific cut. The cross-validation performance found that using shin samples, a correct classification rate of 75.6% was achieved, using rump samples, a correct classification rate of 70.4% could be achieved, and when using the neck samples, the correct classification rate was 72.7%. This is consistent with the earlier findings of modelling performance being improved by greater sample sizes.

## Discussion

REIMS has shown itself to be capable of identifying several key features of beef cuts from a single rapid measurement, it has been demonstrated that the type of cut can be reliably determined with an accuracy greater than 98%, whilst the production system can be determined with an accuracy of around 85%, despite different breeds of cattle being used to develop the models, an approach taken to ensure the models were representative of commercial beef production.

Organic beef production has been extensively studied from a nutritional and environmental viewpoint, but very little work has been published which shows analytical methods being established which can differentiate between organic and conventional beef.

The analytical difficulties in establishing what exactly differentiates organic from conventional beef are better understood, following on from two studies in Ireland that made use of SIRA^12,13^.

Despite it is so difficult for any analytical technique to differentiate between the two production systems, the loading plot for the two class discriminatory model (Fig. 5) shows that when comparing organic and conventional beef using REIMS, a significant number of relatively minor changes in lipid profile occur, suggesting that even relatively minor changes to animal diet and husbandry, particularly exposure to medications, can have a small but detectable effect on the fingerprint generated by the REIMS system.

No individual biomarkers were identified in distinguishing organic and conventional beef, this is largely consistent with the previous discussions concerning similarities in feed, the limited separation observed indicated it is unlikely a single or small group of biomarkers will emerge, rather, discreet differences in the intensities of many or all of the lipid species present are driving the separation witnessed thus far (Fig. 5). The incorporation of an organically produced biomarker that can be detected using REIMS may, however, be worth further consideration.

**Table 3:** Leave-20%-Out cross-validation results for the production system model.

	Number of spectra	Number of passes	Number of failures	Number of outliers	Correct classification rate (excluding outliers)	Correct classification rate (including outliers)
	561	470	90	1	83.9%	83.8%
Group	Conventional	Organic	Outlier	Total		
Conventional	248	53	0	301		
Organic	37	222	1	260		
Total	285	275	1	561		

The presence of veterinary drug residues in meat was believed to provide another meaningful way to differentiate between organic and conventional beef production as there are strict controls on the use of such medications in the organic livestock sector; however, a growing acceptance of the need to reduce veterinary drug use, particular antimicrobials, is resulting in considerably more restrained use even under conventional production systems^25,26^. The use of antimicrobials is particularly strongly regulated in conventional animal production in Europe through Directive 96/22/EC, which can result in conventional animals having little or no difference in their exposure to such compounds, when compared to organically reared animals, so the direct analysis of veterinary compounds and their metabolites no longer represents a robust approach in confirming the authenticity of organic meat. The previously discussed SIRA approaches also seem to be sub-optimal in confirming the authenticity of organically produced meat, despite the advantages it can bring in terms of confirming other aspects of meat authenticity, particularly geographic.

Testing with SIRA is time-consuming due to the sample preparation required. SIRA samples must be cut into thin pieces, freeze-dried, and homogenised using a ball mill before lipids are extracted, and defatted muscle residue is analysed (Schmidt et al., 2005). The performance of SIRA is also highly variable and several areas of concern identified; in a comparable study where it was used to assess production system and diet authenticity, performance ranged from a cross-validation performance of 70.8–100%^27,28^. This variability, cost, and time penalties in analysis for SIRA leaves the technique at a significant disadvantage compared to the reproducibility, low cost, and rapid analysis times demonstrated with REIMS in this study.

The use of REIMS to confirm meat authenticity is further bolstered by its extremely powerful ability to perform other important measurements simultaneously. This research showed a greater than 98% ability to correctly differentiate between different cuts from the same animal, at the same time as being able to provide an assessment on the likely production system.

The misrepresentation of meat cuts is itself a stand-alone crime described as a potential beef fraud by Ballin (2010) due to the price differences between various cuts of meat.

Recent studies have been able to distinguish meat cuts using an electronic nose system (GeNose) with an accuracy of 96%^29^, whilst REIMS was able to distinguish meat samples with a 98% correct classification rate. REIMS would therefore be a viable option for distinguishing meat cuts and detecting this type of substitution fraud. The utility of the system in detecting fraud in higher value cuts will likely form a further package of work in future, following on from the success of the system in identifying lower value cuts.

Lastly, REIMS has no sample preparation, testing only takes a matter of seconds and can be performed by personnel with more basic training. The instrumentation can be situated several metres away from the production line with only minimal equipment needed immediately adjacent to the production line. These factors make REIMS an attractive option for beef industry use.

The technique is presently restricted to expensive time-of-flight high resolution mass spectrometers with substantial maintenance requirements. This could however be resolved by the development of a REIMS ion-source which fits onto an inexpensive, compact and robust single quadrupole mass spectrometer.

This research has determined REIMS is a highly promising technique to determine various aspects of fraud in the beef industry, from organic fraud to substitution of cheap cuts in place of prime cuts.

This technology, if deployed appropriately and the results interpreted as part of a wider quality assurance scheme should be capable of reassuring retailers and consumers that their organic beef is genuine. The application of the REIMS technique also shows increasing promise in identifying different cuts of beef, and can therefore detect multiple types of fraud.

REIMS most useful attribute may be that it does not require any sample preparation, this ease of use enables operation of the system by non-specialised personnel, and may relatively easily be incorporated near to or within a production line environment. The utility and value of such a system in the meat industry could be substantial.

## Methods

### Sample collection

Carcass samples from the neck (supraspinatus), rump (gluteus), and shin (flexor carpi radialis) regions were collected over a nineteen-month period between April 2019 and November 2020. All samples were taken from four abattoirs, located in the Republic of Ireland, Northern Ireland, and England (ABP Cahir, ABP Newry, ABP Ellesmere, and ABP Clones). A combination of organic and conventional samples were taken, with the animals chosen for sampling being taken from trusted farmers to ensure authenticity. Breeds of cattle included Aberdeen Angus, Limousin, Hereford, and Simmental to provide representative samples of a commercial beef production environment in Ireland and the United Kingdom. After slaughter and primary processing of selected animals, carcasses were moved to a refrigerator chiller where they were stored at 2 °C. All samples were then taken three days after the animal had been slaughtered. Each sample was approximately 100–120 g, and samples were cut into three equal pieces, labelled and vacuum packed, then stored at −80 °C. Before undergoing REIMS analysis, samples were thawed to approximately 4 °C. In total 561 samples were collected, 285 of these sample were from conventionally produced cattle and 276 were from organically produced cattle.

### REIMS experimental setup

A Waters REIMS ion-source was connected to a Waters Xevo G2-XS QToF mass spectrometer (Waters, Wilmslow, UK). Meat samples were burned by use of a bipolar probe assembly (Waters Research Centre, Budapest, Hungary) connected to an ERBE VIO 50C diathermy generator (Erbe Elektromedizin, Tubingen, Germany).

A 0.1 ng/µL lockmass solution of leucine enkephalin (Leu-Enk) in 2-propanol (Honeywell Riedel-de Haën, Seelze, Germany) was infused into the REIMS source using a Waters Acquity I-class UPLC system (Waters, Milford, MA, USA) at a rate of 0.2 mL/min to enable accurate mass correction and to assist in the ionisation process within the ion-source.

The mass spectrometer was set to acquire data in continuum mode, at a scan speed of 0.5 s per scan. The mass range was m/z 50–1200 and the total run time per sample was set to 2 min.

The instrument underwent a detector setup process using 0.1 ng/µL Leu-Enk solution in 2-propanol, and then calibration using 5 mM sodium formate infusion (20 µL/min) at the start of each day to compensate for instrument drift and variation.

Meat samples were removed from their packaging and placed on an insulated chopping board, after which they were burned using the bi-polar probe approximately 10 times, with each burn event lasting for between 3 and 5 s. No carry-over effects were observed between samples.

### Data analysis

REIMS data was acquired using Waters MassLynx (SCN 949) (Waters, Wilmslow, UK) and then imported into Waters Abstract Model Builder (AMX) software (v 0.9.2092.0) (Waters Research Centre, Budapest, Hungary). The data was subject to the MassLynx pre-processing and peak picking algorithms, after which it was lockmass corrected to m/z 554.2615 using the Leu-Enk signal contained within each RAW data file, background subtracted and normalised using total ion count (TIC).

The recorded scans for each sample were combined to give an average spectrum, resulting in one averaged spectrum for each sample being used in the subsequent chemometric model building. The chemometric models were generated using a mass range of m/z 600–1000 and spectral threshold intensity of 2e5, mass binning was then performed with the data split into 0.5 Da mass bins.

AMX was used to generate principle component analysis (PCA) and then linear discriminant analysis (LDA) models, with the first PCA models being used to reduce the dimensionality of the data prior to LDA analysis, an approach referred to as PCA-LDA modelling.

AMX enables a leave-20%-out cross-validation of the PCA-LDA score plots. This was undertaken on the PCA-LDA models generated, the standard deviation for this was set to 5σ, with each sample classified into the closest class, or if outside the standard deviation range of 5σ for all classes, then recorded as an outlier.

Models were generated to review both meat cuts and production systems, a three class model being used for the meat cuts work and a binary (two class) model for the production system work. A more complex model with six classes was used to determine whether both cut and production system could be determined simultaneously.

## Data Availability

The authors declare that all relevant data supporting this study has been included within the paper. Raw data will be made available by the corresponding authors upon reasonable request.
